# Effect of aspirin and other non-steroidal anti-inflammatory drugs on prostate cancer incidence and mortality: a systematic review and meta-analysis

**DOI:** 10.1186/1741-7015-12-55

**Published:** 2014-03-28

**Authors:** Yanqiong Liu, Jun-Qiang Chen, Li Xie, Jian Wang, Taijie Li, Yu He, Yong Gao, Xue Qin, Shan Li

**Affiliations:** 1 Department of Clinical Laboratory, First Affiliated Hospital of Guangxi Medical University, Nanning, Guangxi Zhuang Autonomous Region, China; 2 Department of Gastrointestinal Surgery, First Affiliated Hospital of Guangxi Medical University, Nanning, Guangxi Zhuang Autonomous Region, China; 3 General Practice School, Guangxi Medical University, Nanning, Guangxi Zhuang Autonomous Region, China

**Keywords:** Aspirin, NSAID, Prostate cancer, Incidence, Mortality

## Abstract

**Background:**

It has been postulated that non-steroidal anti-inflammatory drugs (NSAIDs) use leads to decreased prostate cancer (PCa) risk. In recent years, NSAIDs’ role in PCa development has been extensively studied; however, there is not yet a definitive answer. Moreover, the epidemiological results for NSAIDs’ effect on PCa-specific mortality have been inconsistent. Therefore, we performed a meta-analysis to examine the controversy.

**Methods:**

We performed a literature database search and included all published studies conducted in the general population exposed to any NSAID, extracting an odds ratio (OR) or a hazard ratio (HR) with 95% confidence intervals (95% CIs) that compared the incidence of PCa or PCa-specific mortality with non-exposure. We derived a pooled OR or HR using random or fixed effects models, as appropriate. Subgroup analyses were also performed.

**Results:**

Thirty-nine studies (20 case–control and 19 cohort studies) were included in this analysis. Thirty-one studies were available concerning NSAID use and PCa incidence and eight studies on PCa-specific mortality. Compared to non-use, aspirin use was statistically significantly associated with PCa incidence risk, and the association was slightly stronger for advanced PCa than for total PCa (OR = 0.92, 95% CI = 0.87 to 0.97 for total PCa; OR = 0.81, 95% CI = 0.73 to 0.89 for advanced PCa). Aspirin use seems also to be associated with a modest reduction in PCa-specific mortality (HR = 0.86, 95% CI = 0.78 to 0.96 for total PCa; OR = 0.81, 95% CI = 0.71 to 0.92 for advanced PCa). Generally, the pooled effects for any NSAIDs, NA-NSAIDs and cyclooxygenase-2 inhibitors demonstrated no adverse or beneficial effects on PCa development or PCa-specific mortality, but the results were not consistent. The effect estimates did not vary markedly when stratified by study design and study quality but varied by geographic region. Furthermore, long-term aspirin use (≥4 years) was also significantly associated with reduced PCa incidence (OR = 0.88, 95% CI 0.79 to 0.99).

**Conclusions:**

The present meta-analysis provides support for the hypothesis that aspirin use is inversely related to PCa incidence and PCa-specific mortality. The effect estimates, varying by geographic region, deserve further investigation.

## Background

Prostate cancer (PCa) is one of the most frequently occurring cancers and cause of cancer-related deaths in men [1]. According to the latest report from the American Cancer Society, in 2014, 233,000 new cases will be diagnosed, and 29,480 estimated deaths from PCa are predicted in the United States [1]. Although the five-year relative survival rate has increased over the past 25 years [2], PCa is still the leading cause of cancer death in older men [1]. There is an urgent need for a better understanding of the factors related to PCa development and prognosis.

Cyclooxygenase-2 (COX-2), an inducible enzyme, is overexpressed in PCa tissue [3] and plays a role in PCa cell growth [4]. Aberrant or high expression of COX-2 has been implicated in carcinogenesis and poorer prognosis [5,6]. Therefore, it has been speculated that non-steroidal anti-inflammatory drugs (NSAIDs), which inhibit the COX pathway, may provide a strategy for mechanistically based PCa chemoprevention and therapy [3]. Indeed, a number of epidemiologic studies have investigated the association between NSAID use and the risk of PCa but showed conflicting results, with the majority finding null effects [7-22], some reporting statistically significant inverse associations [23-34] and a minority reporting a significantly elevated risk of prostate cancer in association with NSAID or non-aspirin NSAID (NA-NSAID) use [35-37]. In a study conducted by Mahmud *et al*. [38], which searched databases in June 2008, the association of the use of aspirin and other NSAIDs with PCa incidence was suggestive but not conclusive. Several observational studies published after this meta-analysis have shown contrasting results [19-22,32-34,36,37], which has added new evidence to the previous research. On the other hand, since considerable evidence implies that aspirin use may reduce the risk of PCa, a better understanding of whether such therapy can influence disease outcome is important and necessary. To date, several studies have examined NSAID (aspirin) use and PCa-specific mortality, and these have reported conflicting findings [39-46].

Given widespread and long-term NSAID use, more knowledge is needed to clarify the drugs’ role in PCa incidence and mortality. Therefore, we performed a comprehensive review and provide a quantitative assessment of all relevant published studies to understand this issue better. A better understanding of the relationship may also highlight the importance of considering additional prevention methods in this area.

## Methods

### Data sources and searches

This study was performed according to the Preferred Reporting Items for Systematic Reviews and Meta-Analyses (PRISMA) checklist [see Additional file 1] [47]. Three authors (YL, JC and LX) experienced in Cochrane review searched electronic databases for articles published through 29 December 2013. The databases included PubMed, Embase, ISI Web of Science and the WHO Library Database. The search terms included the therapeutic classes, generic names of individual drugs and PCa outcome terms [see Additional file 2]. No language restrictions were imposed. Additional studies were searched for manually through the reference lists of retrieved articles and using PubMed’s related articles option.

### Eligibility criteria and study selection

To be included, studies had to meet the following criteria: (1) studied participants were exposed to any NSAID, including aspirin, NA-NSAIDs, any other single NSAID (not including acetaminophen) or a mixture of NSAIDs or selective COX-2 inhibitors and reference participants had not used these drugs; (2) the study assessed the incidence of PCa or PCa-specific mortality; and (3) the study reported the multivariate-adjusted relative risks (RRs), including study-specific odds ratios (ORs) or hazard ratios (HRs) and 95% confidence intervals (CIs). No study design restrictions were imposed. Reviews, letters, comments, lectures and case reports were all excluded. When study populations overlapped, only the study of larger size or the most informative one was included. However, if overlapping studies offered additional information for subgroup analysis that could not be extracted from the primary studies, they were included in the subgroup analysis. Two authors (JW, TL) independently evaluated all records by title and abstract and subsequently retrieved and assessed in detail the full text of any potentially relevant articles according to the eligibility criteria. Disagreements or uncertainties regarding eligibility were resolved through discussion with two additional adjudicators (XQ, SL).

### Data extraction and quality assessment

For each trial, the study and participant characteristics, number of cases and controls, drug types, exposure period and multivariable adjusted RR estimates with corresponding 95% CIs were extracted and transferred to specially designed forms. If the required data for the meta-analysis were not readily available in the published article, the principal author was contacted at least once. Data were obtained and independently reviewed by two reviewers (YL and SL), and the final decision was reached by consensus. The methodological quality of observational studies was assessed by two authors (XQ and YL) independently using the Newcastle-Ottawa scale (NOS) [48]. Disagreement was resolved by consensus. In this scale, studies are awarded a maximum score of 9 points; a high-quality study is awarded ≥7 points, a medium-quality study between 4 and 6 points and a poor-quality study <4 points.

### Data synthesis and statistical analysis

We evaluated the association of NSAID use with two endpoints: (1) the OR of PCa incidence and (2) the HRs of PCa-specific mortality. Pooled ORs and HRs with 95% CI were obtained using a fixed effects model [49] or the random effects model of DerSimonian and Laird [50] if needed due to between-study heterogeneity. Because the evaluated outcomes are relatively rare and the effects estimated are generally small, ORs in case–control studies were considered reasonable approximations of the corresponding risk ratios in cohort studies [51], permitting the combination of cohort and case–control studies. Inter-study heterogeneity was tested by Cochran’s Q test [52] and quantified by the *I*^*2*^ statistics [53]. For the Q statistic, a *P* value <0.10 was considered statistically significant for heterogeneity; for *I*^*2*^, a value >50% indicates a measure of heterogeneity [52]. Cumulative meta-analysis was conducted to examine how the evidence has changed over time. We assessed publication bias graphically using a funnel plot and quantitatively using the Begg rank correlation test and the Egger regression asymmetry test [54,55] where numbers of studies allowed (>10 studies). Sensitivity analyses were also conducted to assess the robustness of the results by the sequential omission of individual studies [56].

To detect potential interactions, studies were stratified by the type of medicine (NSAIDs overall, aspirin, NA-NSAIDs and COX-2 inhibitors) and study outcome (total PCa (all cancers regardless of stage), advanced cancers (lesions with extracapsular extension or metastases to regional lymph nodes or other organs) and non-advanced cancers). Further subgroup analysis estimated the effects of NSAIDs on PCa risk by study design, studies defined as high quality, geographic region and duration of aspirin use. A two-tailed *P* value <0.05 was considered statistically significant. All analyses were performed using STATA version 12.0 (StataCorp, College Station, TX, USA).

## Results

### Study selection, characteristics and quality

**Figure 1:**
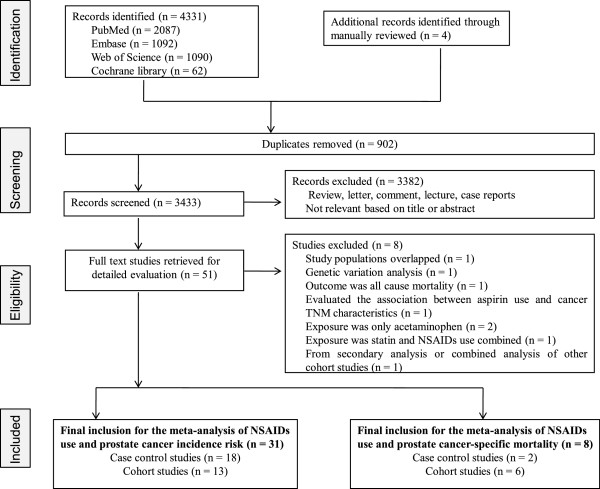
Flow of selection for studies through review.

Our initial search identified 4,331 studies, of which we obtained 51 full-text articles, and 39 studies [7-37,39-46] were included in the review. References for studies excluded from the full-text study review process and the additional records identified through a manual review are listed in Additional file 3. A PRISMA trial flow diagram for systematic review is presented in Figure 1. One article from the Cancer Prevention Study II Nutrition Cohort by Jacobs *et al*. [18] in 2007 was excluded from the primary outcome analysis because this article only updated the duration-effect of aspirin contrasting the primary group [14]; therefore, the study data were re-entered for subgroup analyses for duration of aspirin.

The studies that met the inclusion criteria were all observational studies, comprising 20 case–control [9-11,16,17,21-23,26,28-33,35-37,41],[44] and 19 cohort studies [7,8,12-15,18-20,24,25,27,34],[39,40,42,43,45,46] involving more than 924,502 male subjects, including 108,136 PCa cases. Thirty-one studies (18 case–control [9-11,16,17,21-23,26,28-33,35-37] and 13 cohort [7,8,12-15,18-20,24,25,27,34]) addressed the use of any NSAID and its association with PCa incidence risk (Table 1); eight studies (two case–control [41,44] and six cohort [39,40,42,43,45,46]) investigated whether NSAID use was associated with PCa-specific mortality (Table 2). For NSAID use and PCa incidence risk, the publication dates of the studies ranged between 1989 and 2014. A majority of the studies were conducted in North America (16 in the USA and four in Canada) and 10 in Europe. For NSAID use and PCa-specific mortality, five studies [39-41,43,45] measured exposure to aspirin, one study [44] addressed any NSAIDs, one study [46] covered only NA-NSAIDs, and one study [42] employed all three categories.

**Table 1:** Characteristics of included studies: any NSAIDs use and PCa incidence risk

**Study**	**Study location**	**Cases (number)**	**Controls (number)**	**Age (y)**^**a**^	**Exposure period**	**Drugs studied**	**Outcome (number)**	**Information source**	**Confounders for adjustment**	**Quality score**
**Case–control studies**										
Veitonmaki, 2013 [37]	Finland	24,657	24,657	68	1995–2002	ASA/NSAIDs/ NA-NSAIDs/ COX-2 inhibitors	Total/advanced PCa	Database	1–5	8
Kopp, 2013 [22]	Denmark	334	334	59.0	1993–1997	NSAIDs	Total PCa	Questionnaire	1, 6, 7	4
Vinogradova, 2011 [21]	UK	14,764	192,081	69	1997–2008	COX-2 inhibitors	Total PCa	Database	1, 8–11	7
Murad, 2011 [36]	UK	1,016	5,043	63	2001–2008	ASA/NSAIDs/ NA-NSAIDs	Total PCa	Questionnaire	1, 12–15	6
Mahmud, 2011 [33]	Canada	9,007	36,028	73	1985–2000	ASA/NSAIDs/ NA-NSAIDs	Total PCa	Database	1, 14–17	6
Salinas, 2010 [32]	USA	1,001	942	63	2002–2005	ASA/ NA-NSAIDs/ COX-2 inhibitors	Total/advanced PCa	Questionnaire	1, 17–18	7
Harris, 2007 [31]	USA	229	285	NR	2002–2004	ASA/ NA-NSAIDs/ COX-2 inhibitors	Total PCa	Interview	1, 6, 19–21	5
Menezes, 2006 [17]	USA	1,029	1,029	67	1982–1998	ASA	Total/advanced PCa	Questionnaire	1, 6, 12	4
Mahmud, 2006 [30]	Canada	494	805	64	1999–2003	ASA/ NSAIDs/ NA-NSAIDs/ COX-2 inhibitors	Total/advanced PCa	Questionnaire	1, 12, 14, 15, 22–25	6
Liu, 2006 [29]	USA	506	506	NR	2001–2004	ASA/ NSAIDs / NA-NSAIDs	Advanced PCa	Interview	1, 18, 26	5
Dasgupta, 2006 [28]	Canada	2,025	2,150	73	1999–2002	ASA/ NA-NSAIDs	Total PCa	Database	1, 27	6
Bosetti, 2006 [16]	Italy	1,261	1,131	65	1991–2002	ASA	Total/advanced PCa	Questionnaire	1, 7, 12, 28	5
Perron, 2003 [26]	Canada	2,221	11,105	75.7	1993–1995	ASA/ NA-NSAIDs	Total PCa	Database	1, 29	6
Irani, 2002 [11]	France	639	659	66.8	1999–2000	ASA/ NA-NSAIDs	Total PCa	Questionnaire	1, 14, 15, 18, 27, 30–32	6
Nelson, 2000 [23]	USA	417	420	64.0	1992–1995	NA-NSAIDs/ NSAIDs	Total PCa	Interview	1, 18, 23	6
Langman, 2000 [35]	UK	1,813	5,354	NR	1993–1995	NSAIDs	Total PCa	Database	1, 9	6
Norrish, 1998 [10]	New Zealand	317	480	70.0	1996	ASA/ NSAIDs/ NA-NSAIDs	Total/advanced PCa	Questionnaire	1, 33–36	7
Neugut, 1998 [9]	USA	319	189	69.0	1984–1986	ASA	Total PCa	Medical notes	1, 18, 13, 22	6
**Cohort studies**			**All subjects (number)**		**Median follow-up (y)**					
Shebl, 2012 [34]	USA	3,573	29,450	62.8	11.7	ASA/NA-NSAIDs	Total/advanced PCa	Questionnaire	1, 12, 14, 15, 17, 18, 28	6
Dhillon, 2011 [20]	USA	4,858	51,529	64.8	18.0	ASA	Total/advanced PCa	Questionnaire	1, 4, 6, 9, 12, 18, 20, 31, 36–39	8
Brasky, 2010 [19]	USA	1,550	34,132	50–76	NR	ASA/ NA-NSAIDs	Total/advanced PCa	Questionnaire and Database	1, 6, 7, 12, 13, 18, 22, 39–45	5
Jacobs, 2007 [18]	USA	1,076	69,810	NR	104,854 person-years	ASA	Total PCa	Questionnaire and medical records	1, 6, 7, 9, 13, 15, 18, 20, 22, 45–47	7
Platz, 2005 [15]	USA	141	1,244	70.0	9.0	ASA/NSAIDs/ NA-NSAIDs	Total PCa	Questionnaire	1, 14, 15, 23, 37,	7
Jacobs, 2005 [14]	USA	4,853	70,144	NR	1992–2001	ASA/NSAIDs/ NA-NSAIDs	Total/advanced PCa	Questionnaire	1, 7, 12, 13, 18, 22, 45,	7
G-Rodriguez, 2004 [27]	UK	2,183	12,183	50–79	1995–2001	ASA/NA-NSAIDs	Total/advanced PCa	Database	1, 14, 15, 17, 37, 48	8
Sorensen, 2003 [13]	Denmark	324	172,057	NR	5.4	NA-NSAIDs	Total PCa	Database	1, 10, 11	7
Friis, 2003 [12]	Denmark	196	29,470	70.0	4.1	ASA	Total PCa	Database	1, 10, 11	7
Roberts, 2002 [25]	USA	91	1,362	64.0	5.5	NSAIDs	Total PCa	Questionnaire	1, 10, 12, 20	7
Habel, 2002 [24]	USA	2,574	90,100	18–84	14.0	ASA	Total/advanced PCa	Questionnaire	1, 7, 17, 18,	6
Schreinemachers, 1994 [8]	USA	123	12,668	65.0	12.4	ASA	Total PCa	Interview	1, 7, 9, 18, 21	5
Paganini-Hill, 1989 [7]	USA	149	5106	73	6.5	ASA	Total PCa	Questionnaire	1	3

^a^average, median or range.Confounders for adjustment: 1, age; 2, benign prostatic hyperplasia medication use; 3, anti-diabetic medication; 4, cholesterol-lowering medication use; 5, antihypertensive medication use; 6, body mass index; 7, school education; 8, deprivation; 9, smoking; 10, comorbidities; 11, use of medication; 12, family history of prostate cancer; 13, diabetes status; 14, aspirin use; 15, any NA-NSAID use; 16, ever visited a urologist 1 to 11 years prior; 17, screened and volume of family physician visits; 18, race; 19, family history; 20, physical activity; 21, alcohol intake; 22, history of heart disease; 23, intake of acetaminophen; 24, reasons for referral and prostate volume; 25, selective cyclooxygenase-2 (COX-2) inhibitors use; 26, medical institution; 27, finasteride use; 28, study center; 29, recent medical contacts; 30, farming; 31, frequency of red meat and red wine consumption; 32, urological center; 33, socio-economic status; 34, total polyunsaturated fat consumption; 35, α-linolenic acid; 36, fatty acids; 37, period; 38, height; 39, vitamin; 40, osteoarthritis; 41, rheumatoid arthritis; 42, chronic joint pain; 43, chronic headaches; 44, migraines; 45, PSA test in the past two years; 46, history of colorectal endoscopy; 47, hypertension; 48, prior benign prostate hyperplasia history. ASA, aspirin; COX-2, cyclooxygenase enzymes-2; NA-NSAIDs, non-aspirin NSAIDs; NR, not reported; PCa, prostate cancer; y, years.

**Table 2:** Characteristics of included studies: any NSAIDs use and prostate cancer-specific mortality

**Study**	**Study location**	**Study design**	**PCa Cases (number)**	**Died from PCa (number)**	**Age (y)**	**Median follow-up (y)**	**Drugs studied**	**Treatment**	**Tumor stage**	**Confounders for adjustment**	**Quality score**
Flahavan, 2014 [39]	Ireland	Cohort	2,936	104	70.5	5.5	Aspirin	PR, RT, ADT	I–III	1–8	8
Grytli, 2014 [40]	Norway	Cohort	3,561	1,010	76.3	3.3	Aspirin	ADT	I–IV	1, 2, 9–13	7
Cardwell, 2013 [41]	UK	Nested case–control	6,339	1,184	NR	6.0	Aspirin	PR, RT, ADT, EST	I–IV	1, 4, 8, 13–17	7
Dhillon, 2012 [42]	USA	Cohort	3,986	265	68.6	8.4	Aspirin/NSAIDs/ NA-NSAIDs	PR, RT, EST	I–IIIab	1, 2, 4, 6, 10, 18–29	7
Choe, 2012 [43]	USA	Cohort	5,955	193	64.0	5.8	Aspirin	RP, RT	I–IV	1, 2, 9, 10, 29–31	8
Stock, 2008 [44]	Canada	Case–control	1,619	453	67.2	NR	NSAIDs	RP, RT	I–IV	1, 2, 9, 21	7
Ratnasinghe, 2004 [45]	USA	Cohort	NR	121	25–74	NR	Aspirin	NR	NR	1, 4, 19, 21, 32–33	6
Lipworth, 2004 [46]	Denmark	Cohort	NR	296	48.4	4.3	NA-NSAIDs	NR	NR	1, 33	5

Confounders for adjustment: 1, age; 2, tumor grade; 3, tumor size; 4, smoking status; 5, co-morbidity score; 6, year of incidence; 7, pre-diagnostic statin exposure 8, receipt of radiation; 9, prostate-specific antigen level; 10, Gleason score; 11, presence and type of metastases; 12, performance status; 13, androgen deprivation therapy initiated within six months after diagnosis; 14, year of cancer diagnosis; 15, chemotherapy within six months of diagnosis; 16, estrogen therapy during exposure period; 17, comorbidities; 18, family history; 19, race; 20, height; 21, body mass index; 22, vigorous physical activity; 23, vitamin D; 24, fish; 25, red meat; 26, cholesterol-lowering drugs; 27, total kcal; 28, aspirin use before diagnosis; 29, initial treatment; 30, aspirin use; 31, non-aspirin anticoagulant use; 32, poverty index; 33, education; 33, number of prescriptions. ADT, androgen-deprivation therapy; EST, estrogen therapy; NA-NSAIDs, non-aspirin NSAIDs; NR: not reported; RP, radical prostatectomy; RT, radiation therapy; y, years.

With regard to the quality of all the included studies assessed by NOS (Table 1 and Table 2), 18 studies (46.2%) were graded as having good quality (seven or more points), 20 (51.3%) were graded as medium-quality (four to six points), and one study (2.5%) conducted in 1989 was graded as poor-quality (three points). The details of the quality assessment are presented in Additional file 4.

### Association between the use of any NSAID and the incidence of prostate cancer

**Figure 2:**
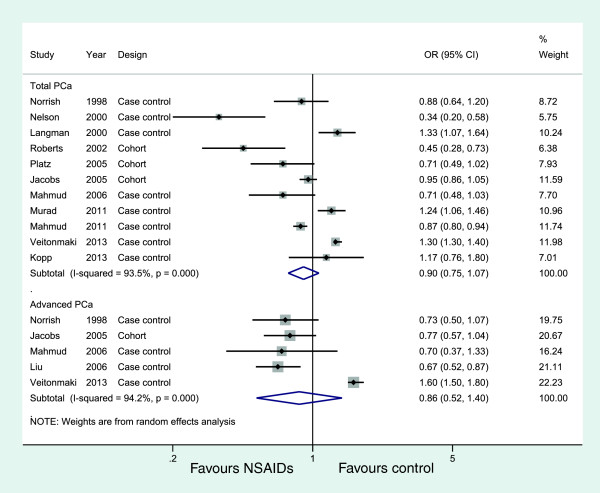
Association between use of any NSAIDs and incidence of prostate cancer.

Eleven studies (eight case-controls and three cohorts) evaluated exposure to any NSAIDs and the incidence of total PCa, and five studies examined the effect of any NSAIDs on the incidence of advanced PCa, but no studies examined non-advanced PCa. Figure 2 shows the study-specific and pooled ORs and 95% CIs of PCa for any NSAID users versus non-NSAID users. The summary OR for the 11 studies that assessed the effect of any NSAIDs on total PCa was 0.90 (95% CI: 0.75 to 1.07; *I*^*2*^ = 93.5%). The effect estimates were similar for five studies on any NSAIDs and advanced PCa (OR = 0.86, 95% CI: 0.52 to 1.40) (Figure 2, Table 3). These analyses yielded substantial heterogeneity (*I*^*2*^ = 93.5% and *I*^*2*^ = 94.2%, respectively) and revealed that any NSAIDs were not associated with a significant decrease in the incidence of PCa.

**Table 3:** Results of subgroup analyses by outcome type and NSAIDs type

**Study characteristics**	**Number of studies**	**OR (95% CI)**	***P***_***OR***_**value**	**Effect model**	**Heterogeneity**	
					***I***^***2***^ *** (%)***	***P*** **value**
**Risk of prostate cancer incidence**						
**Any NSAIDs**						
**Studies of total prostate cancer**	11	0.90 (0.75, 1.07)	0.25	Random	93.5	<0.001
Case–control studies	8	0.97 (0.79, 1.20)	0.78	Random	93.8	<0.001
Cohort studies	3	0.71 (0.47, 1.07)	0.10	Random	81.4	0.005
Studies in North America	6	0.73 (0.60, 0.88)	**0.001**	Random	79.6	<0.001
Studies in Europe	5	1.29 (1.25, 1.34)	**<0.001**	Fixed	37.2	0.17
**Studies of advanced prostate cancer**	5	0.86 (0.52, 1.40)	0.54	Random	94.2	<0.001
**Aspirin**						
**Studies of total prostate cancer**	23	0.92 (0.87, 0.97)	**0.002**	Random	66.2	<0.001
Case–control studies	13	0.92 (0.85, 0.99)	**0.040**	Random	63.7	0.001
Cohort studies	10	0.91 (0.83, 0.99)	**0.040**	Random	71.7	<0.001
Studies from North America	16	0.92 (0.86, 0.97)	**0.003**	Random	53.7	0.006
Studies from Europe	7	0.94 (0.82, 1.08)	0.40	Random	80.7	<0.001
High quality studies	8	0.89 (0.81, 0.98)	**0.022**	Random	77.2	<0.001
Long-term aspirin use (≥4 years)	8	0.88 (0.79, 0.99)	**0.038**	Random	66.8	0.004
**Studies of advanced prostate cancer**	13	0.81 (0.73, 0.89)	**<0.001**	Fixed	23.9	0.20
Case–control studies	7	0.84 (0.73, 0.98)	**0.025**	Fixed	23.7	0.18
Cohort studies	6	0.77 (0.67, 0.89)	**<0.001**	Fixed	23.5	0.26
Studies from North America	9	0.82 (0.75, 0.89)	**<0.001**	Fixed	14.0	0.32
Studies from Europe	4	0.88 (0.75, 1.03)	1.22	Fixed	48.2	0.12
High quality studies	6	0.81 (0.72, 0.92)	**0.002**	Fixed	38.1	0.152
**Studies of non-advanced prostate cancer**	6	0.96 (0.87, 1.07)	0.460	Fixed	33.6	0.177
**Non-aspirin NSAIDs**						
**Studies of total prostate cancer**	17	1.01 (0.90, 1.13)	0.86	Random	90.1	<0.001
Case–control studies	11	0.97 (0.81, 1.17)	0.74	Random	93.0	<0.001
Cohort studies	6	1.07 (0.95, 1.20)	0.25	Random	69.6	0.006
Studies from North America	11	0.94 (0.85, 1.05)	0.28	Random	68.8	<0.001
Studies from Europe	6	1.18 (1.06, 1.32)	**0.002**	Random	71.1	0.004
**Studies of advanced prostate cancer**	9	0.99 (0.77, 1.28)	0.97	Random	81.6	<0.001
**Studies of non-advanced prostate cancer**	3	1.00 (0.90, 1.12)	0.943	Fixed	0.0	0.897
**Any COX2 inhibitors**						
**Studies of total prostate cancer**	5	1.10 (0.90, 1.33)	0.36	Random	48.7	0.099
**Studies of advanced prostate cancer**	3	1.20 (0.79, 1.83)	0.40	Fixed	0.0	0.87
**Risk of prostate cancer-specific mortality**						
**Any NSAIDs (including aspirin use alone)**						
**Studies of total prostate cancer**	8	1.00 (0.68, 1.47)	0.99	Random	95.3	<0.001
**Aspirin**						
**Studies of total prostate cancer**	6	0.86 (0.78, 0.96)	**0.005**	Fixed	39.2	0.15
Studies from North America	3	0.85 (0.50, 144)	0.55	Random	63.8	0.063
Studies from Europe	3	0.85 (0.76, 0.95)	**0.005**	Fixed	12.8	0.32

CI, confidence interval; COX-2, cyclooxygenase enzymes-2; OR, odds ratio.

In sensitivity analyses, none of the individual studies substantially altered the pooled ORs for any NSAIDs on total PCa, which varied from 0.86 (95% CI, 0.71 to 1.04) to 0.96 (95% CI, 0.81 to 1.14). The direction and magnitude of the negative effect did not vary markedly when stratified by study design (Table 3). However, when we combined studies by geographic region, the six studies from North America had a summary OR of 0.73 (95% CI: 0.60 to 0.88; *P*_*OR*_ =0.001; *I*^*2*^ = 79.6%), whereas the other five studies from Europe had a higher and statistically significant summary OR of 1.29 (95% CI: 1.25 to 1.34; *P*_*OR*_ <0.001; *I*^*2*^ = 37.2%) (Table 3).

**Figure 3:**
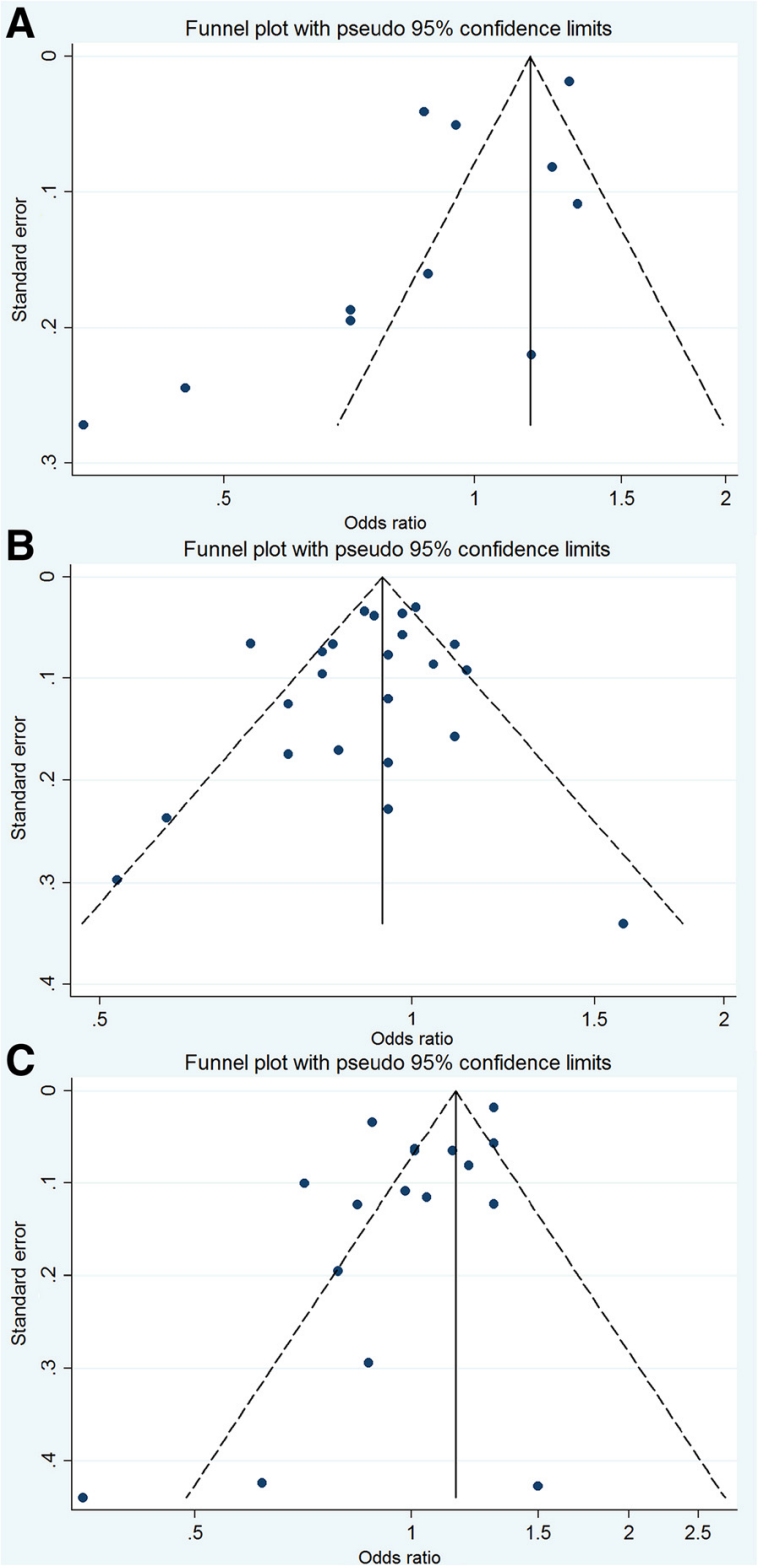
**Funnel plots of the relative risk of total prostate cancer incidence. (A)** for any NSAIDs use; **(B)** for aspirin use; **(C)** for non-aspirin NSAID use.

For publication bias assessment, the inverted funnel plot was potentially asymmetric, and the results of the Begg test (*P* = 0.350) and the Egger test (*P* = 0.037) implied some evidence of publication bias (Figure 3A).

### Association between aspirin use and PCa incidence

**Figure 4:**
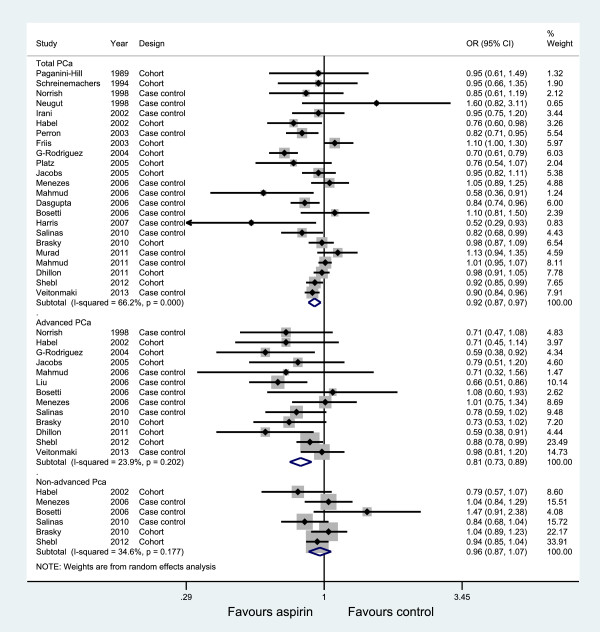
Association between aspirin use and incidence of prostate cancer.

Twenty-three studies, thirteen studies, and six studies evaluated exposure to aspirin and the incidence of total PCa, advanced PCa, and non-advanced PCa, respectively. Figure 4 graphs the ORs and 95% CIs from the individual studies and the pooled results. The 23 studies that assessed the effect of aspirin on total PCa showed an inverse association (OR = 0.92, 95% CI: 0.87 to 0.97) and were moderately heterogeneous (*I*^*2*^ = 66.2%, *P*<0.001). The 13 studies of advanced PCa were consistent; the risk decrease persisted and was slightly lower than for the total PCa (OR = 0.81, 95% CI: 0.73 to 0.89; *I*^*2*^ = 23.9%). However, a non-significant decreased risk was observed in non-advanced PCa (OR = 0.96, 95% CI: 0.87 to 1.07), with little evidence of heterogeneity (*I*^*2*^ = 34.6%, *P* = 0.177).

We saw no evidence of obvious influential studies in sensitivity analyses by the sequential omission of individual studies. The summary ORs for total cancer were reasonably stable, ranging from 0.91 when the study by Friis *et al*. [12] was excluded to 0.94 when the study by Rodriguez *et al*. [27] was excluded. Table 3 shows the results of the subgroup analyses. The risk decrease persisted for both total and advanced PCa when the data were stratified into subgroups based on study design and study quality. The effect sizes showed moderate statistical heterogeneity among studies of total PCa but no heterogeneity among studies of advanced PCa. Adjustment for geographic region had the strongest influence on the summary OR, demonstrating negative results.

We examined the association between long-term aspirin use (defined as ever use of aspirin for more than four years) and risk of total PCa incidence using the data available from the included studies. Eight studies [15,17-20,26,27,32] were included in this analysis. The pooled results slightly strengthened the association of aspirin use with total PCa (OR: 0.88, 95% CI: 0.79, 0.99) (see Table 3 and Additional file 5: Figure S1). Most studies lacked information on the dose and frequency of aspirin use, or the information varied in each trial; hence, a statistical analysis of significance between these groups was not valid.

A visual inspection of the funnel plot found no evidence of publication bias (Figure 3B). The *P* values for the Begg test and the Egger test were *P* = 0.316 and *P* = 0.273, respectively, both suggesting a very low probability of publication bias.

A cumulative meta-analysis of a total of 23 studies of total PCa was carried out to evaluate the cumulative effect estimate over time. In 1989, Paganini-Hill *et al*. [7] first reported a non-significant effect estimate of 0.95. Between 1994 and 2006, 14 studies were published, with a cumulative OR of 0.89 (95% CI: 0.81 to 0.98). A statistically significant effect of aspirin use on PCa incidence was consistently observed after publication of the 13th trial in 2006. Between 2007 and 2013, eight more publications were added cumulatively, resulting in an overall effect estimate of 0.92 (95% CI 0.87 to 0.97) [see Additional file 5: Figure S2].

### Association between NA-NSAID use and PCa incidence

**Figure 5:**
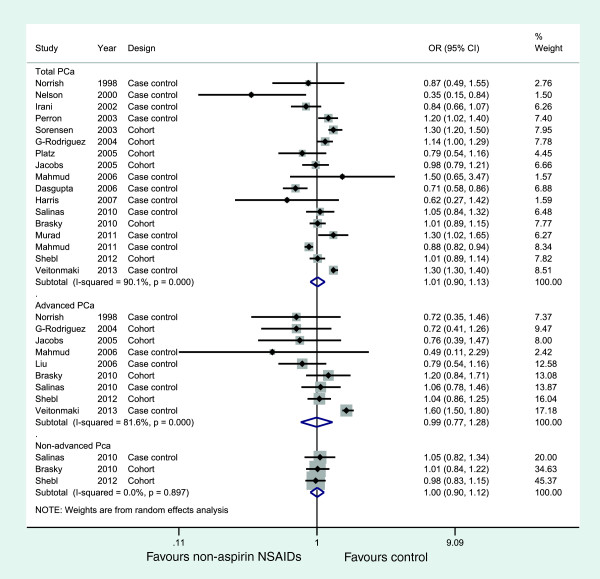
Association between non-aspirin NSAID use and incidence of prostate cancer.

Seventeen studies, nine studies, and three studies evaluated exposure to NA-NSAID and the incidence of total PCa, advanced PCa, and non-advanced PCa, respectively. Figure 5 illustrates the ORs and 95% CIs from the individual studies and the pooled results. The meta-analysis revealed that NA-NSAID use was not significantly associated with the incidence of PCa (OR = 1.01, 95% CI: 0.90 to 1.13 for total cancer; OR = 0.99, 95% CI: 0.77 to 1.28 for advanced cancer; OR = 1.00, 95% CI: (0.90 to 1.12 for non-advanced cancer), both with substantial heterogeneity between studies except for non-advanced PCa analysis. Table 2 presents the results of subgroup analyses.

For publication bias, the funnel plot was slightly asymmetric (Figure 3C), and the *P* values for the Begg test and the Egger test were 0.711 and 0.050, respectively, suggesting a low probability of publication bias.

### Association between any COX-2 inhibitor use and PCa incidence

Five studies [21,30-32,37] evaluated COX-2 inhibitor use and PCa risk. Overall, use of COX-2 inhibitors appeared not to be statistically significantly associated with PCa risk compared to non-use (Table 3; Additional file 5: Figure S3). For studies with small numbers, we did not perform subgroup analyses.

### Association between any NSAID use and PCa-specific mortality

**Figure 6:**
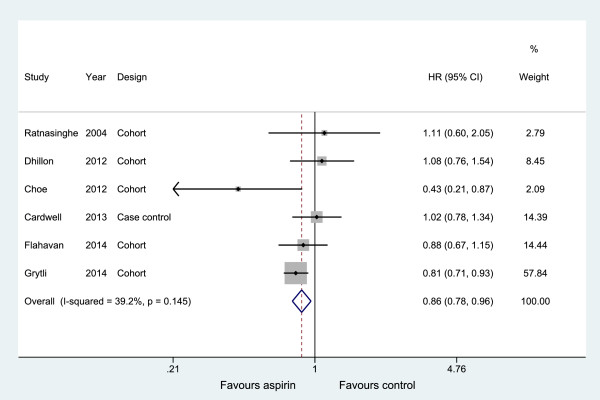
Association between aspirin use and prostate cancer-specific mortality.

Eight studies [39-46] investigated exposure to any NSAIDs use (including aspirin use alone) with PCa-specific mortality (Table 2). The pooled result shows that the use of any NSAID had a neutral influence on the PCa-specific mortality in a random effects model, with an HR = 1.00 (95% CI: 0.68, 1.47) but with substantial heterogeneity (*I*^*2*^ = 95.3%) (Table 3 and Additional file 5: Figure S4). Studies of aspirin were more consistent; the pooled results indicated that aspirin use was statistically significantly associated with a modest reduction in PCa-specific mortality (OR = 0.86, 95% CI: 0.78 to 0.96), with little evidence of heterogeneity (Table 3 and Figure 6). Confined to three studies from North America, the HR was 0.85 (95% CI: 0.50 to 144) but with more substantial heterogeneity (*I*^*2*^ = 6%).

## Discussion

This meta-analysis, involving a total of 108,136 PCa cases from 39 observational studies, examined the association of the use of any NSAID with the risk of PCa incidence or mortality. The major finding of the present meta-analysis provides support for the mechanistic hypothesis that aspirin use (including long-term use of more than four years) is inversely related to the risk of PCa incidence. The association was slightly stronger for advanced PCa than for total PCa (OR = 0.92 for total PCa, OR = 0.81 for advanced PCa). A cumulative meta-analysis showed that a statistically significant effect of aspirin use on PCa incidence was consistently observed after publication of the 13th trial in 2006. More importantly, aspirin use demonstrated a 14% decrease in PCa-specific mortality for total PCa compared to non-use. In general, the pooled effects for any NSAIDs, NA-NSAIDs and COX-2 inhibitors demonstrated no adverse or beneficial effects on PCa development or PCa-specific survival, but the results were not consistent; all the effect estimates varied by geographic region.

There is a long-standing debate about the relationship between NSAID use and cancer. Several reviewed studies discussed the potential chemoprevention effects of NSAID use against tumors at various sites, including breast [57], lung [58] and brain [59], and melanoma [60]. This meta-analysis, with a much larger number of participants than previous meta-analyses [38,61], adds to the previous findings by showing that aspirin use demonstrates a protective effect against PCa. With a larger sample size, increased statistical power could be obtained. In addition, we evaluated whether the association varied by COX-2 inhibitor, long-term aspirin use and study quality, and we investigated whether NSAID use influenced mortality from the disease, issues that were not discussed in the primary meta-analysis. Moreover, the present studies included an approved quality evaluation system; thus, it was more reliable in minimizing potential bias.

There are several proposed mechanisms by which these effects could be explained. Primary laboratory studies have pointed to COX-2 overexpression in PCa tissue [3], and it is well established that increased expression of COX-2 is related to tumor growth [4] and poorer prognosis [5]. NSAIDs inhibit PCa cell growth by reducing the synthesis of prostaglandins by COX enzymes [62], including inducing apoptosis [63], inhibiting cellular proliferation and angiogenesis [64]. Animal and laboratory studies have confirmed the preventive effect of NSAIDs on PCa repeatedly and consistently. In a review of 12 studies, the authors reported that, in all the included studies, all types of NSAIDs exhibited inhibitory effects on PCa development and progression.

In our study, use of aspirin was significantly inversely related to the risks of developing advanced PCa but not non-advanced PCa, which is difficult to explain. It may possibly be explained by detection bias if aspirin users were more likely to be screened and, therefore, to be diagnosed at an earlier stage. However, this effect may be real. Laboratory studies have suggested that advanced stage PCa may represent heterogeneous etiologies [65] and the mechanism by which aspirin acts on the initiation of poorly-differentiated disease may be distinct from its influence on the development of advanced disease [20]. There was also evidence that NSAIDs evoke tumor regression in gastrointestinal tumors [66], and may have similar effects against cancers of other sites including the prostate. Our findings with regard to the effects of aspirin on disease severity require confirmation in larger studies using unbiased diagnostic approaches.

We postulated that the use of aspirin reduced overall PCa risk. This could have significant implications with respect to the dose, frequency and duration of aspirin use. Unfortunately, most studies lacked information on dosage, frequency and duration of exposure. Although some studies have provided this information, they varied in each trial, resulting in invalid statistical analysis in these groups. In a study by Veitonmaki *et al*. [37] in 2013, a dose–effect relationship was found, indicating a significant inverse association (OR = 0.83) only among participants who used aspirin at the dosage of 37 to 1,300 defined daily dose (DDD). The other benefit was shown among those who took more than one aspirin pill per day [23,29,34], a low dose (≤75 mg/daily) [27,32], larger doses (≥325 mg/daily) [26] or more than six tablets/week [20]; nevertheless, other studies found no evidence of a dose–effect [7,10,17,19,33,35] or frequency–effect [14,16,17] relationship. In terms of the duration of aspirin use, we used the available data from eight studies [15,17-20,26,27,32] with durations of more than four years. Our pooled results demonstrated a negative trend of PCa risk with more than four years of aspirin use.

Considering the high incidence of PCa and the widespread use of aspirin in the general population, therefore, successful prevention could have a major public health impact. However, physicians should be aware of which subgroups of the population are at high risk for PCa, and the optimal dosage of aspirin, as well as its side effects, should be addressed. It seems that the use of aspirin in clinical prevention still has a long way to go, but, at least, the evidence implies that it is beneficial against the risk of PCa.

Nevertheless, several limitations of this study must be acknowledged. First, heterogeneity was a potential problem when interpreting the results of our analysis. In our meta-analysis, significant heterogeneity was observed in many of the analyses we conducted. Despite stratifying the data into subgroups based on type of outcome, study design, geographic region, quality of study and duration of aspirin use, some heterogeneity was still detected. In fact, it is no surprise given the differences of each study in the definition of drug exposure, information collection methods (for example, questionnaires or medical records), race, age and lifestyle factors of participants, sample size, duration of study follow-up and so on.

Second, as shown in Table 1 and Table 2, the number and content of the adjusted confounders differed among studies, which could have caused some imprecision in the effect estimates. The established risk factors for PCa are African-American ethnicity and family history of PCa [65]. Most studies adjusted for age and race using multivariate statistical models. Few studies adjusted for family history of PCa. However, researchers do not always make the same decisions concerning confounding factors. We did our best to minimize these confounding biases by choosing the most multivariable adjusted-effect estimates to analyze.

In addition, several sources of bias could have affected our observed associations. First, observational studies have inherent limitations, notably bias and unmeasured confounding. On the other hand, screening and surveillance biases are potentially a major source of systemic error in the reviewed studies. NSAID use may be associated with the socioeconomic status of patients. Thus, NSAID users might maintain healthier lifestyle habits than non-users, which could influence their risk for PCa. Moreover, NSAID users may have easier and more frequent access to preventive healthcare services, such as screening for prostate-specific antigen (PSA), which theoretically could lower the incidence of PCa.

At this stage, randomized controlled trials (RCTs) would be required to evaluate further the relationship between NSAIDs and PCa risk. However, RCTs for this topic could be extremely difficult or even impossible. First, PCa is rare and mainly occurs in older men; second, too many people in the general population take NSAIDs, especially aspirin, for various important medical reasons. This will render randomization and adherence to the protocol impossible. If one wants to conduct such a trial in younger subjects who do not take NSAIDS, investigators will run into the trouble of having enough end-points (events) given their young age.

## Conclusions

Our current study, based on the available studies and updated data from a previous meta-analysis, supports the hypothesis that aspirin use (including long-term use) provides potential benefits in the reduction of PCa incidence and PCa-specific mortality. The inverse association was slightly stronger for advanced PCa than for total PCa, but the effect estimates varied by geographic region. Also unclear is the influence of dose and the frequency of aspirin use on PCa incidence and outcomes. Thus, caution needs to be exercised to ensure that the associated prevention benefits of aspirin outweigh the potential side effects (for example. gastrointestinal bleeding).

## Abbreviations

ASA: aspirin; CI: confidence interval; COX-2: cyclooxygenase enzymes-2; HR: hazard ratio; NA-NSAIDs: non-aspirin nonsteroidal anti-inflammatory drugs; NOS: Newcastle-Ottawa scale; NSAIDs: nonsteroidal anti-inflammatory drugs; OR: odds ratio; PCa: prostate cancer; RCT: randomized controlled trial; RR: relative risk.

## Competing interests

The authors declare that they have no competing interests.

## Authors’ contributions

YL, JC and LX conducted the literature search and quality assessment and contributed to the drafting of the manuscript. YL, JW and TL performed data extraction and statistical analysis. YH consulted on statistical analysis. JC and YG critically revised drafts of the manuscript. QX and SL conceived and designed the review, assisted with the inclusion/exclusion criteria, acted as independent assessors and critically revised drafts of the manuscript. All of the authors contributed to the interpretation of data and all of the authors critically revised the manuscript. All of the authors are guarantors for the study. All of the authors read and approved the final manuscript.

## Pre-publication history

The pre-publication history for this paper can be accessed here:

http://www.biomedcentral.com/1741-7015/12/55/prepub

## Supplementary Material

Additional file 1 PRISMA 2009 Checklist. Click here for file

Additional file 2 Review methodology. Click here for file

Additional file 3 Excluded studies and manually reviewed publications. Click here for file

Additional file 4 NOS scores. Click here for file

Additional file 5: Figure S1 Association between long-term aspirin use and incidence of total prostate cancer. **Figure S2.** Cumulative meta-analysis of 23 studies on the association of aspirin use and total prostate cancer incidence risk. **Figure S3.** Association between any COX-2 inhibitor use and incidence of prostate cancer. **Figure S4.** Association between any NSAIDs use and prostate cancer-specific mortality. Click here for file
