# Supplementary material for: Effect of aspirin and other non-steroidal anti-inflammatory drugs on prostate cancer incidence and mortality: a systematic review and meta-analysis
Source: BMC Med. 2014 Mar 28;12:55. doi: 10.1186/1741-7015-12-55 (PMC4021622; doi:10.1186/1741-7015-12-55)
Supplement: Additional file 2 — Review methodology. [file 1741-7015-12-55-S2.pdf]

Additional file 2

Review methodology

**Electronic databases**	PubMed, Embase, ISI Web of Science, and WHO Library Database
**Search terms #1**	anti-inflammatory agents OR non-steroidal anti-inflammatory drugs OR NSAID* OR selective non-steroidal anti-inflammatory drugs OR non-selective non-steroidal anti-inflammatory drugs OR cyclo-oxygenase-2 inhibitors OR cox-2 inhibitors OR selective cyclo-oxygenase-2 inhibitors OR selective cox-2 inhibitors OR aspirin OR acetylsalicylic acid OR celecoxib OR rofecoxib OR meloxicam OR lumiracoxib OR valdecoxib OR parecoxib OR etoricoxib OR diclofenac OR ibuprofen OR naproxen OR piroxicam OR indomethacin OR indometacin OR azapropazone OR etodolac OR fenbufen OR fenoprofen OR flurbiprofen OR ketoprofen OR ketorolac OR mefenamic acid OR nabumetone OR sulindac OR tenoxicam OR tiaprofenic acid
**Search terms #2**	prostate carcinoma OR prostate cancer OR prostatic neoplasms
**Search terms #3**	Search terms #1 AND search terms #2
