# Supplementary material for: Effect of aspirin and other non-steroidal anti-inflammatory drugs on prostate cancer incidence and mortality: a systematic review and meta-analysis
Source: BMC Med. 2014 Mar 28;12:55. doi: 10.1186/1741-7015-12-55 (PMC4021622; doi:10.1186/1741-7015-12-55)
Supplement: Additional file 3 — Excluded studies and manually reviewed publications. [file 1741-7015-12-55-S3.pdf]

Additional file 3

Text S1 References for studies excluded from the full text studies review

<u>Excluded Studies</u>: categorized by reasons for exclusion

Study populations overlapped

1. Leitzmann MF, Stampfer MJ, Ma J, Chan JM, Colditz GA, Willett WC, Giovannucci E: **Aspirin use in relation to risk of prostate cancer**. *Cancer Epidemiol Biomarkers Prev* 2002, **11**(10 Pt 1):1108-1111.

Genetic variation analysis

1. Cheng I, Liu X, Plummer SJ, Krumroy LM, Casey G, Witte JS: **COX2 genetic variation, NSAIDs, and advanced prostate cancer risk**. *Br J Cancer* 2007, **97**(4):557-561.

Outcome was all cause mortality

1. Katz MS, Carroll PR, Cowan JE, Chan JM, D'Amico AV: **Association of statin and nonsteroidal anti-inflammatory drug use with prostate cancer outcomes: results from CaPSURE**. *BJU Int* 2010, **106**(5):627-632.

Evaluated the association between aspirin use and cancer TNM characteristics

1. Jonsson F, Yin L, Lundholm C, Smedby KE, Czene K, Pawitan Y: **Low-dose aspirin use and cancer characteristics: a population-based cohort study**. *Br J Cancer* 2013, **109**(7):1921-1925.

Exposure was only acetaminophen

1. Walter RB, Brasky TM, White E: **Cancer risk associated with long-term use of acetaminophen in the prospective VITamins and lifestyle (VITAL) study**. *Cancer Epidemiol Biomarkers Prev* 2011, **20**(12):2637-2641.

2. Jacobs EJ, Newton CC, Stevens VL, Gapstur SM: **A large cohort study of long-term acetaminophen use and prostate cancer incidence**. *Cancer Epidemiol Biomarkers Prev* 2011, **20**(7):1322-1328.

Exposure was statin and NSAIDs use combined

1. Coogan PF, Kelly JP, Strom BL, Rosenberg L: **Statin and NSAID use and prostate cancer risk**. *Pharmacoepidemiol Drug Saf* 2010, **19**(7):752-755.

From secondary analysis or combined analysis of other cohort studies

1. Rothwell PM, Wilson M, Price JF, Belch JFF, Meade TW, Mehta Z: **Effect of daily aspirin on risk of cancer metastasis: a study of incident cancers during randomised controlled trials**. *Lancet* 2012, **379**(9826):1591-1601.

Text S2 Additional publications related to included studies

1. Paganini-Hill A, Chao A, Ross RK, Henderson BE: **Aspirin use and chronic diseases: a cohort study of the elderly**. *Bmj* 1989, **299**(6710):1247-1250.

2. Schreinemachers DM, Everson RB: **Aspirin use and lung, colon, and breast cancer incidence in a prospective study**. *Epidemiology* 1994, **5**(2):138-146.

3. Ratnasinghe LD, Graubard BI, Kahle L, Tangrea JA, Taylor PR, Hawk E: **Aspirin use and mortality from cancer in a prospective cohort study**. *Anticancer Res* 2004, **24**(5B):3177-3184.

4. Vinogradova Y, Coupland C, Hippisley-Cox J: **Exposure to cyclooxygenase-2 inhibitors and risk of cancer: nested case-control studies**. *Br J Cancer* 2011, **105**(3):452-459.

.
