# Supplementary material for: Effect of aspirin and other non-steroidal anti-inflammatory drugs on prostate cancer incidence and mortality: a systematic review and meta-analysis
Source: BMC Med. 2014 Mar 28;12:55. doi: 10.1186/1741-7015-12-55 (PMC4021622; doi:10.1186/1741-7015-12-55)
Supplement: Additional file 4 — NOS scores. [file 1741-7015-12-55-S4.pdf]

Additional file 4

Table S1 NOS scores of case control studies

NOS scale	Veitonmaki 2013	Kopp 2013	Vinogradova 2011	Murad 2011	Mahmud 2011	Salinas 2010	Harris 2007	Menezes 2006	Mahmud 2006	Liu 2006
**A Selection (maximum 4)**	**4**	**3**	**4**	**4**	**3**	**4**	**3**	**2**	**3**	**3**
1 Case definition adequate	1	0	1	1	0	1	1	0	1	1
2 Representativeness of the cases	1	1	1	1	1	1	1	1	1	1
3 Selection of controls	1	1	1	1	1	1	0	0	0	0
4 Definition of controls	1	1	1	1	1	1	1	1	1	1
**B Comparability (maximum 2)**	**2**	**0**	**1**	**1**	**1**	**1**	**1**	**1**	**2**	**1**
1 Comparability of cases and controls on the basis of the design or analysis	2	0	1	1	1	1	1	1	2	1
**C Exposure (maximum 3)**	**2**	**1**	**2**	**1**	**2**	**2**	**1**	**1**	**1**	**1**
1 Ascertainment of exposure	1	0	1	0	1	0	0	0	0	0
2 Same method cases and controls?	1	1	1	1	1	1	1	1	1	1
3 Non-response rate	0	0	0	0	0	1	0	0	0	0
**Totaal (maximum 9)**	**8**	**4**	**7**	**6**	**6**	**7**	**5**	**4**	**6**	**5**

NOS scale	Dasgupta 2006	Bosetti 2006	Perron 2003	Irani 2002	Nelson 2000	Langman 2000	Norris 1998	Neugut 1998	Cardwell 2013	Stock 2008
**A Selection (maximum 4)**	**3**	**3**	**3**	**3**	**4**	**3**	**4**	**3**	**3**	**4**
1 Case definition adequate	1	1	0	1	1	0	1	1	0	1
2 Representativeness of the cases	1	1	1	1	1	1	1	1	1	1
3 Selection of controls	0	0	1	0	1	1	1	0	1	1
4 Definition of controls	1	1	1	1	1	1	1	1	1	1
**B Comparability (maximum 2)**	**1**	**1**	**1**	**2**	**0**	**1**	**1**	**1**	**2**	**1**
1 Comparability of cases and controls on the basis of the design or analysis	1	1	1	2	0	1	1	1	2	1
**C Exposure (maximum 3)**	**2**	**1**	**2**	**1**	**2**	**2**	**2**	**2**	**2**	**2**
1 Ascertainment of exposure	1	0	1	0	1	1	0	1	1	1
2 Same method cases and controls?	1	1	1	1	1	1	1	1	1	1
3 Non-response rate	0	0	0	0	0	0	1	0	0	0
**Totaal (maximum 9)**	**6**	**5**	**6**	**6**	**6**	**6**	**7**	**6**	**7**	**7**

Table S2 NOS scores of cohort studies

NOS scale	Shebl 2012	Dhillon 2011	Brasky 2010	Jacobs 2007	Platz 2005	Jacobs 2005	Garcia Rodriguez 2004	Sorensen 2003	Friis 2003	Roberts 2002	Habel 2002
**A Selection (maximum 4)**	**3**	**3**	**2**	**3**	**4**	**3**	**4**	**4**	**4**	**4**	**3**
1 Representativeness of the exposed cohort	1	1	1	1	1	1	1	1	1	1	1
2 Selection of the non exposed cohort	1	1	0	1	1	1	1	1	1	1	1
3 Ascertainment of exposure	0	0	0	0	1	0	1	1	1	0	0
4 Demonstration that outcome of interest was not present at start of study	1	1	1	1	1	1	1	1	1	1	1
**B Comparability (maximum 2)**	**1**	**2**	**2**	**2**	**1**	**2**	**2**	**1**	**1**	**1**	**1**
1 Comparability of cohorts on the basis of the design of analysis	1	2	2	2	1	2	2	1	1	1	1
**C Outcome (maximum 3)**	**2**	**3**	**1**	**2**	**2**	**2**	**2**	**2**	**2**	**2**	**2**
1 Assessment of outcome	1	1	1	1	1	1	1	1	1	1	1
2 Was follow-up long enough for outcomes to occur	1	1	0	1	1	1	1	1	1	1	1
3 Adequacy of follow up of cohorts	0	1	0	0	0	0	0	0	0	0	0
**Totaal (maximum 9)**	**6**	**8**	**5**	**7**	**7**	**7**	**8**	**7**	**7**	**7**	**6**

NOS scale	Schreinemachers 1994	Paganini-Hill 1989	Flahavan 2014	Grytli 2013	Dhillon 2012	Choe 2012	Katz 2010	Ratnasinghe 2004	Lipworth 2004
**A Selection (maximum 4)**	**3**	**2**	**4**	**4**	**3**	**4**	**3**	**3**	**3**
1 Representativeness of the exposed cohort	1	1	1	1	1	1	1	1	1
2 Selection of the non exposed cohort	1	1	1	1	1	1	1	1	0
3 Ascertainment of exposure	1	0	1	1	0	1	0	0	1
4 Demonstration that outcome of interest was not present at start of study	0	0	1	1	1	1	1	1	1
**B Comparability (maximum 2)**	**0**	**0**	**2**	**2**	**2**	**2**	**1**	**1**	**0**
1 Comparability of cohorts on the basis of the design of analysis	0	0	2	2	2	2	1	1	0
**C Outcome (maximum 3)**	**2**	**1**	**2**	**1**	**2**	**2**	**2**	**2**	**2**
1 Assessment of outcome	1	0	1	1	1	1	1	1	1
2 Was follow-up long enough for outcomes to occur	1	1	1	0	1	1	1	0	1
3 Adequacy of follow up of cohorts	0	0	0	1	0	0	0	1	0
**Totaal (maximum 9)**	**5**	**3**	**8**	**7**	**7**	**8**	**6**	**6**	**5**
