# Supplementary material for: Effect of aspirin and other non-steroidal anti-inflammatory drugs on prostate cancer incidence and mortality: a systematic review and meta-analysis
Source: BMC Med. 2014 Mar 28;12:55. doi: 10.1186/1741-7015-12-55 (PMC4021622; doi:10.1186/1741-7015-12-55)
Supplement: Additional file 5: Figure S1 — Association between long-term aspirin use and incidence of total prostate cancer. Figure S2. Cumulative meta-analysis of 23 studies on the association of aspirin use and total prostate cancer incidence risk. Figure S3. Association between any COX-2 inhibitor use and incidence of prostate cancer. Figure S4. Association between any NSAIDs use and prostate cancer-specific mortality. [file 1741-7015-12-55-S5.pdf]

Additional file 5

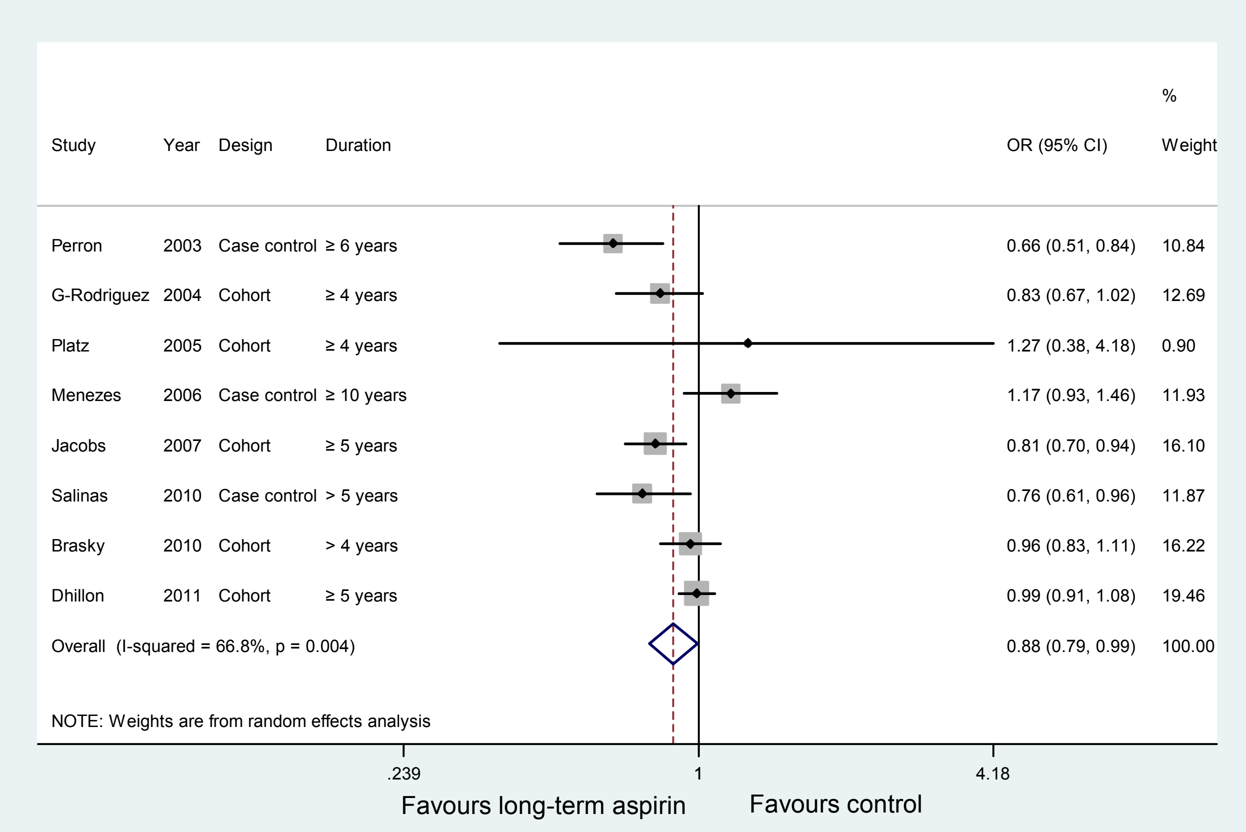

Figure S1 Association between long-term aspirin use and incidence of total prostate cancer.

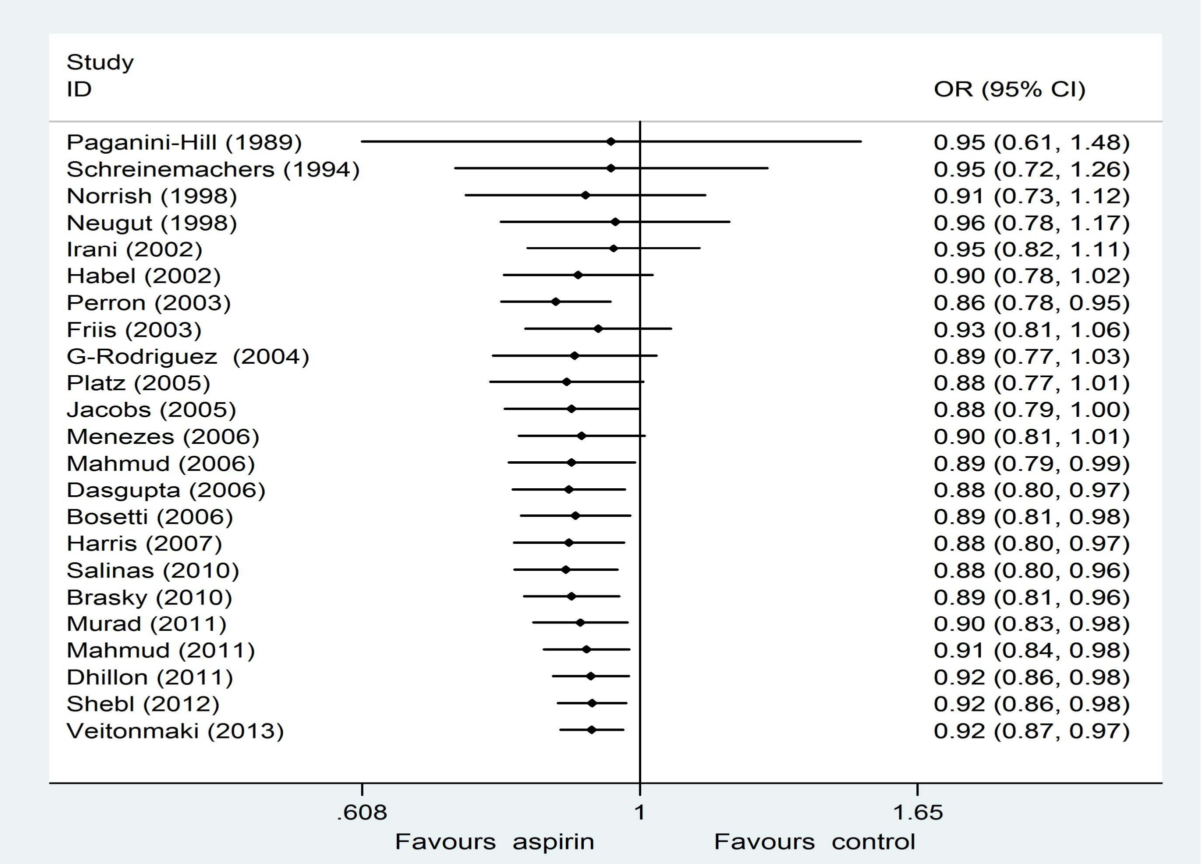

Figure S2 Cumulative meta-analysis of 23 studies on the association of aspirin use and total prostate cancer incidence risk.

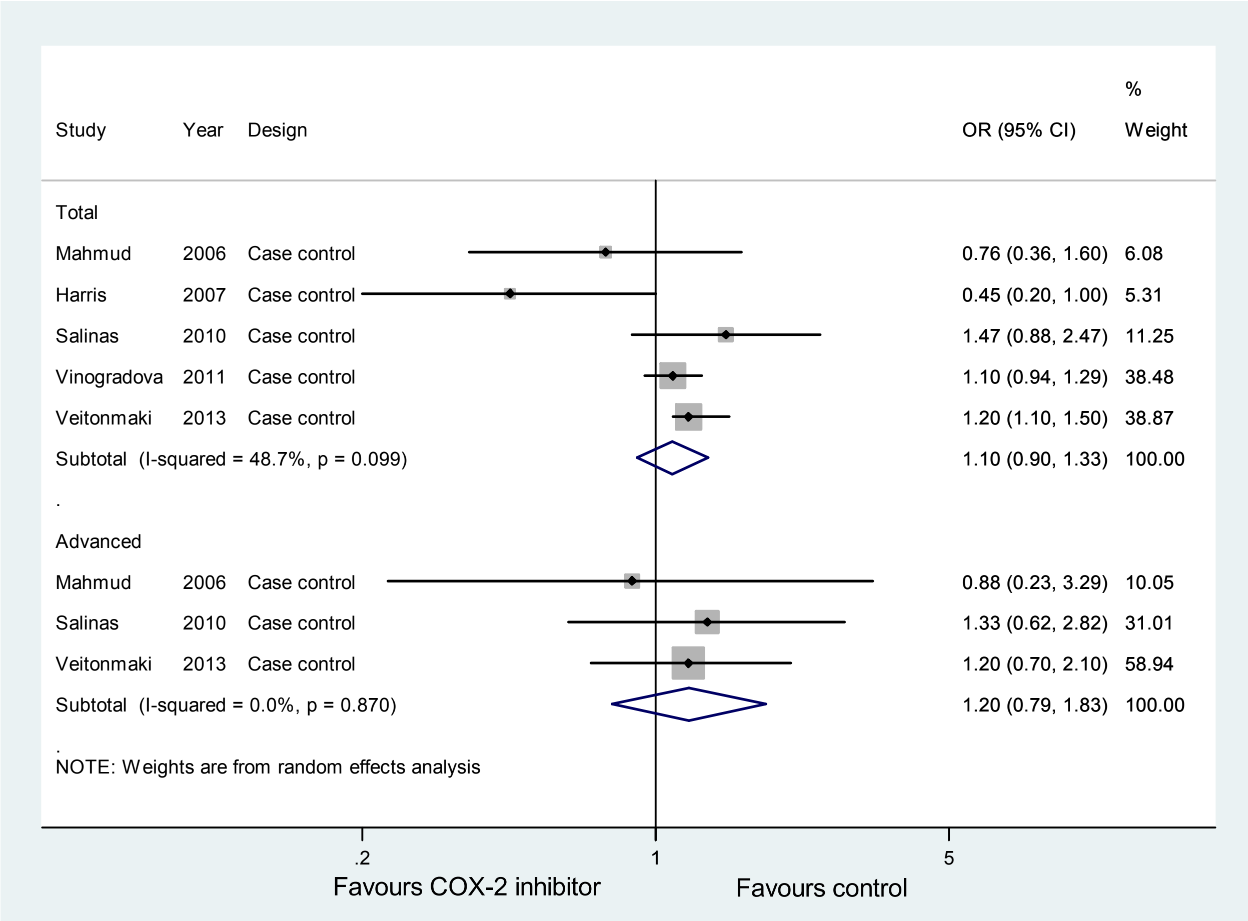

Figure S3 Association between any COX-2 inhibitor use and incidence of prostate cancer.

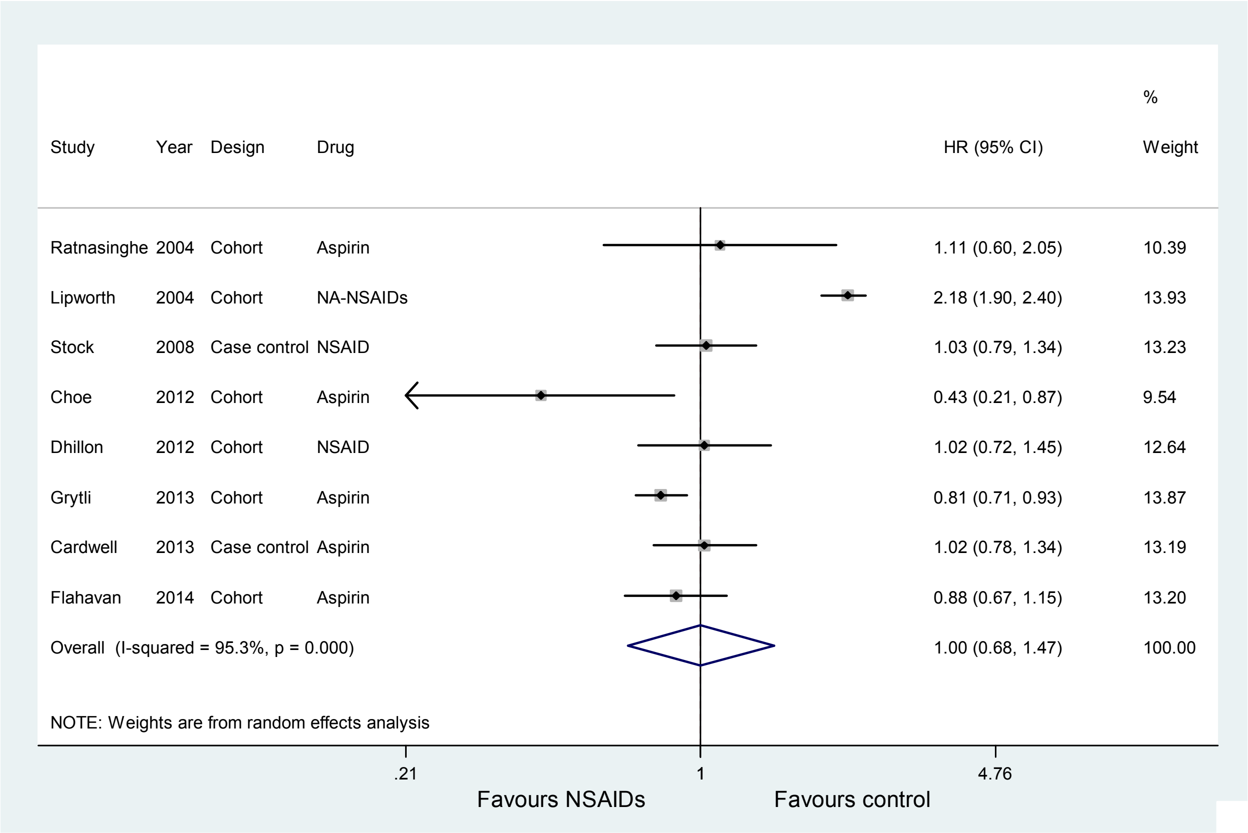

Figure S4 Association between any NSAIDs use and prostate cancer-specific mortality.
